# Two new species and one new record of *Parakiefferiella* Thienemann, 1936 from China (Diptera, Chironomidae)

**DOI:** 10.3897/zookeys.545.6000

**Published:** 2015-12-14

**Authors:** Wenbin Liu, Bingjiao Sun, Chuncai Yan, Chao Song, Xinhua Wang

**Affiliations:** 1 College of Life Science, Nankai University, Tianjin 300071, China; 2 Tianjin Key Laboratory of Animal and Plant Resistance, Tianjin Normal University, Tianjin, 300387, China

**Keywords:** Chironomidae, *Parakiefferiella*, new species, key, taxonomy

## Abstract

Two new species of the genus *Parakiefferiella* Thienemann, 1936 (*Parakiefferiella fasciata* Liu & Wang, **sp. n.** and *Parakiefferiella liupanensis* Liu & Wang, **sp. n.**) are described as adult males from China. *Parakiefferiella tamatriangulata* Sasa is recorded from China for the first time. A key to the known adult males of *Parakiefferiella* from China is presented. The generic diagnosis of male *Parakiefferiella* given by Cranston et al. (1989) is emended.

## Introduction

The genus *Parakiefferiella* was erected by Thienemann in 1936, with *Parakiefferiella coronata* (Edwards, 1929) as type species. According to Cranston et al. (1989), it can be separated from other orthoclad genera by the following combination of characters: eyes bare, without dorsomedial extension; acrostichals absent but scutum with median tuft of microtrichia, sometimes on hump; wing membrane without setae, with fine punctation; squama bare; transverse sternapodeme gently curved, with developed oral projections. Virga strongly developed. Gonostylus with more or less well pronounced curve or bend and slight or absent crista dorsalis. Larval stages of the genus are found in both running and standing waters. Adults fly during spring and summer, rarely in large numbers.

The genus is probably quite speciose. Tuiskunen (1986) described four new species and redescribed eight species from Fennoscandian region. Moubayed (1991, 1994) and recently Moubayed-Breil and Langton (2004) described three additional species: *Parakiefferiella pyrenaica* Moubayed, 1991 (France); *Parakiefferiella wuelkeri* Moubayed, 1994 (western Europe and north Africa); *Parakiefferiella normandiana* Moubayed-Breil & Langton, 2004 (France, Germany and England). According to the catalog of Japanese Orthocladiinae (Yamamoto 2004), ten valid species were record in Japan. Nine species were reported in the Russian Far East by Makarchenko and Makarchenko (2010). To date, 44 species of the genus have been recorded worldwide, of which 33 are Palaearctic, seven are Nearctic, three are Oriental, four are Neotropical and four are Afrotropical (Ashe and O’Connor 2012; Ree, Nam and Jeong 2012).

So far, three species of the genus (*Parakiefferiella bathophila* (Kieffer, 1912), *Parakiefferiella coronata* (Edwards, 1929) and *Parakiefferiella tipuliformis* (Tokunaga, 1940)) were recorded in China (Wang 2000). The species *Parakiefferiella tipuliformis* (Tokunaga, 1940) from Taiwan province previously placed in Spaniotoma (Smittia) by Tokunaga (1940) was transferred to *Parakiefferiella* by Sasa and Kikuchi (1995). The species diagnostic characters fit the genus *Parakiefferiella*. Moreover, *Parakiefferiella coronata* (Edwards, 1929) was recorded just as larval stage (Wang 2000).

Based on specimens from China, two new species are described in this paper, and a key to the species of *Parakiefferiella* in China is provided.

## Materials and methods

Morphology and terminology follow Sæther (1980). The material examined was slide-mounted following the procedures outlined by Sæther (1969). Measurements are given as the range followed by the mean. Color is described as observed in specimen preserved in alcohol. The specimens examined in this study are deposited in the College of Life Sciences, Nankai University, China (BDN).

## Taxonomy

### Parakiefferiella

Taxon classificationAnimaliaDipteraChironomidae

Thienemann, 1936

#### Emended diagnosis.

Based on the material examined and references, the generic diagnosis of male *Parakiefferiella* (Cranston et al. 1989) must be emended as follows: antenna with 12–13 or occasionally with five (*Parakiefferiella gynocera* (Edwards, 1937)) or ten (*Parakiefferiella fasciata* Liu & Wang, sp. n. and *Parakiefferiella liupanensis* Liu & Wang, sp. n.) flagellomeres; wing anal lobe weakly to moderately developed (*Parakiefferiella bathophila* (Kieffer, 1912)); R and R_1_ with few setae, occasionally all veins bare (*Parakiefferiella fasciata* Liu & Wang, sp. n. and *Parakiefferiella liupanensis* Liu & Wang, sp. n.); anal point with 2–8 basal setae, sometimes bare (*Parakiefferiella fasciata* Liu & Wang, sp. n.).

### Parakiefferiella fasciata

Taxon classificationAnimaliaDipteraChironomidae

Liu & Wang sp. n.

http://zoobank.org/4FC0DE0C-3F8D-4746-938E-DBD5E5188AB6

Figs 1–7

#### Type material.

Holotype: ♂ (BDN. No.1165), China, Shandong Province, Yantai City, Kunyu Mountain, 37°30'10"N, 121°23'40"E, 24.viii.1987, sweeping net, Wang XH. The specimens were sealed with Canada balsam on slides. Paratypes: 3 ♂♂, data as holotype.

#### Diagnosis.

The adult male can be distinguished from known species of the genus by the following combination of characters: anal point lacking keel, very broad at base, rounded apically, without basal setae; virga consisting of four spines; antenna with ten flagellomeres, AR 0.47–0.50; tergites III and VIII dark brown, others tergites pale yellow; HR 1.98–2.06; HV 2.65–3.00.

#### Description.

Male imago (n = 4). Total length 1.04–1.09, 1.07 mm. Wing length 0.71–0.72, 0.71 mm. Total length/wing length 1.45–1.56, 1.47. Wing length/length of profemur 3.24–3.29, 3.26.

*Coloration.* Head brown. Thorax brown with dark spot. Tergites (Fig. 4) III and VIII dark brown, others tergites pale yellow.

**Figures 1–7.**
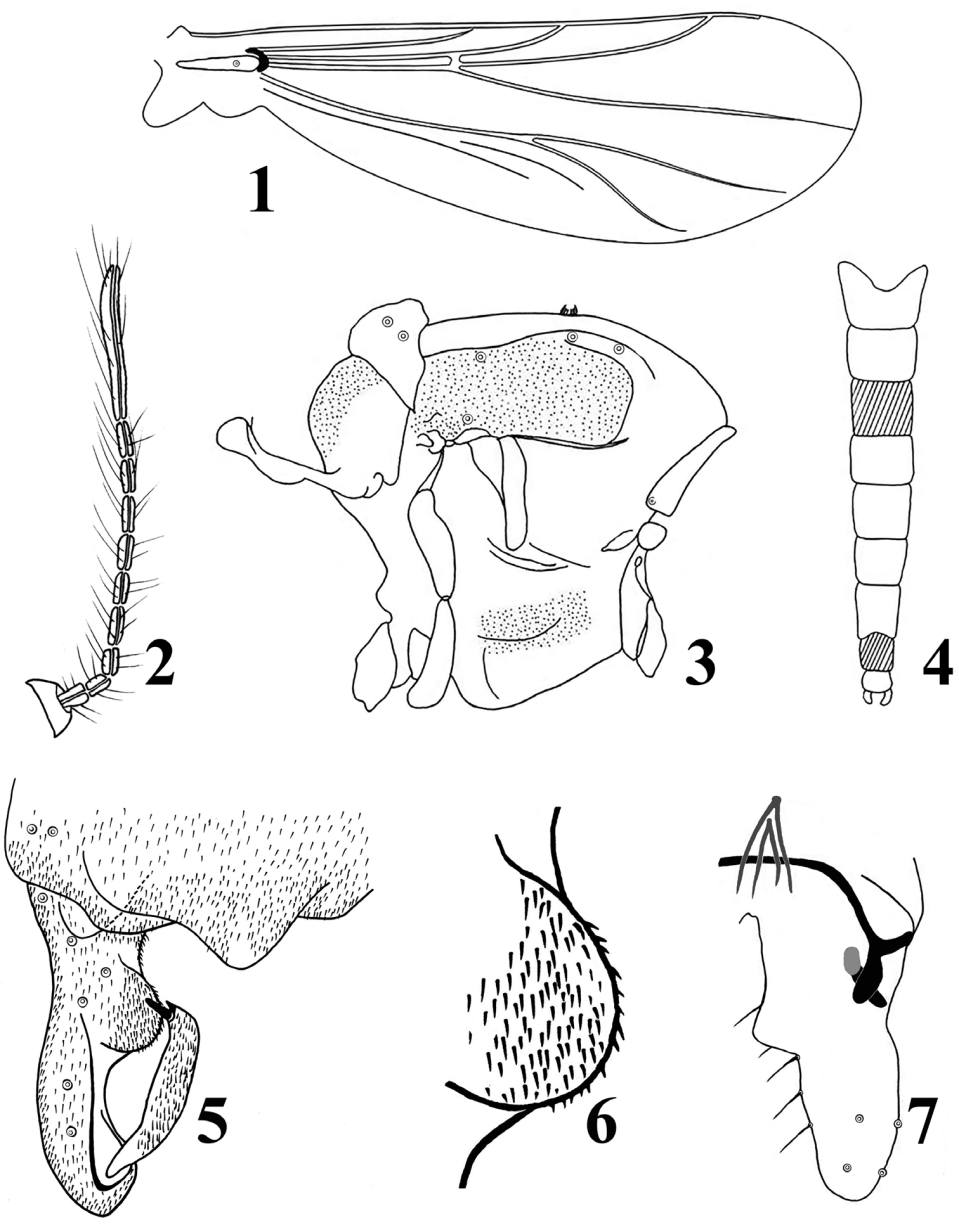
*Parakiefferiella fasciata* Liu & Wang, sp. n., male. **1** wing **2** antenna **3** thorax **4** abdomen tergites coloration **5** hypopygium (dorsal view) **6** inferior; **7** hypopygium (ventral view).

*Head.* Antenna (Fig. 2) with ten flagellomeres. AR 0.47–0.50, 0.49. Ultimate flagellomere 137–144, 140 μm long. Inner vertical 1. Clypeus with four setae. Tentorium 55–72, 66 μm long, 5–7, 6 μm wide. Palpomere lengths (in μm): 10–12, 11; 14–19, 16; 26–29, 28; 43–48, 47; 88–96, 92. L: 5^th^/3^rd ^: 3.26–3.55, 3.43

*Wing* (Fig. 1). Anal lobe reduced. VR 1.12–1.21, 1.17. Costal extension 40 μm long. Brachiolum with one seta. All veins bare. Squama bare.

*Thorax* (Fig. 3). Antepronotal lobes developed. Antepronotum with one lateral seta. Dorsocentrals 2–4, 3; one prealars. Scutellum with 2–6, 5 setae. Mesonotum with a tuft of hairs.

*Legs*. Spur of fore tibia 24–25, 24 μm long; spurs of mid tibia 9–12, 11 μm and 12–14, 13 μm long; spurs of hind tibia 9–10, 9 μm and 20–22, 21 μm long. Hind tibial comb with 9–12, 11 spines, 19–38, 30 μm long. Width at apex of fore tibia 17–19, 19 mm, of mid tibia 19–20, 19 mm, of hind tibia 21–24, 23 mm. Lengths (in μm) and proportions of legs as in Table 1.

**Table 1.** Lengths (in μm) and proportions of legs of *Parakiefferiella fasciata* Liu & Wang, sp. n. (n = 4)

	P_1_	P_2_	P_3_
fe	216–220, 219	288–300, 293	240–258, 256
ti	247–264, 254	240–249, 243	276–282, 278
ta_1_	120–130, 121	108–120, 112	124–139, 131
ta_2_	57–67, 59	57–67, 62	60–68, 66
ta_3_	48–52, 49	43–48, 47	67–76, 71
ta_4_	29–31, 30	29–31, 30	40–43, 41
ta_5_	31–36, 34	30–33, 32	33–38, 34
LR	0.45–0.52, 0.48	0.41–0.44, 0.43	0.45–0.51, 0.48
BV	3.33–3.53, 3.43	3.61–3.87, 3.73	3.07–3.10, 3.09
SV	3.86–4.00, 3.98	4.89–5.00, 4.90	3.88–4.13, 4.01
BR	2.50–2.75, 2.71	3.08–3.25, 3.12	3.50–3.75, 3.54

*Hypopygium* (Figs 5–7). Anal point triangular and hyaline, very broad at base, with rounded apex and without basal seta. Laterosernite IX with 2–4, 3 setae. Phallapodeme 20–24, 23 μm long. Transverse sternapodeme 48–54, 53 μm long. Virga consisting of four spines, 20 –35, 25 μm long. Tergites III and VIII bearing colored bands. Gonocoxite 72–74, 73 μm long, outer margins concave medially. Superior volsella absent; inferior volsella rounded and very large, occupying nearly half of the total length of gonocoxite, lacking dorsal setae and densely covered with microtrichia (Fig. 6); ventral margin strongly swollen medially. Gonostylus 36–38, 37 μm long, anterior margin nearly straight, posterior margin slightly curved in dorsal view. Megaseta 5–7, 7 μm long. Crista dorsalis reduced, slightly visible in lateral view. HR 1.98–2.06, 2.01. HV 2.65–3.00, 2.75.

#### Etymology.

The specific name is from Latin *fasciata*, referring to different colored bands in its tergites.

#### Remarks.

The new described species is distinguished from all other *Parakiefferiella* species by the following unusual combination of characters: antenna with ten flagellomeres; tergites III and VIII with colored bands; anal point lacking both dorsal setae and keel; absence of dorsal setae on inferior volsella, which is bearing only microtrichia.

While the new species close related to *Parakiefferiella coronata* (Edwards, 1929; Makarchenko and Makarchenko 2010). However, the new species differs from the latter on the basis of following characters: (1) the antenna of the new species with ten flagellomeres, whereas *Parakiefferiella coronata* has 13 flagellomeres; (2) the superior volsella of *Parakiefferiella coronata* is large, whereas in the new species it is absent; (3) the new species has abdomen pale yellow with tergites III-VIII dark brown, whereas *Parakiefferiella coronata* has abdomen dark with tergites IV, VI-VII shiny.

Female and immature stages unknown.

### Parakiefferiella liupanensis

Taxon classificationAnimaliaDipteraChironomidae

Liu & Wang sp. n.

http://zoobank.org/816E951D-6283-4643-9640-C9EDC141A97E

Figs 8–13

#### Type material.

Holotype: ♂ (BDN. No.1168), China, Ningxia Hui Autonomous Region, Liupan Mountain, Erlong river, 35°38'40"N, 106°31'40"E, 6.viii.1987, sweeping method, Wang XH. Paratypes: 4 ♂♂, as holotype.

#### Diagnosis.

The adult male can be distinguished from known species of the genus by the following combination of characters: anal point obtuse triangular with pointed and hyaline apex, lacking keel and bearing four setae placed laterally (two on each side); antenna with ten flagellomeres; all of the veins bare; inferior volsella square with rounded inner margin, not projected, occupying about half of the gonocoxite length, lacking dorsal setae and covered with microtrichia; ventral margin swollen medially; AR 0.33–0.37; HR 2.20–2.28; HV 2.36–2.43.

#### Description.

Male imago (n = 5). Total length 1.20–1.25, 1.23 mm. Wing length 0.78–0.85, 0.81 mm. Total length/wing length 1.45–1.47, 1.46. Wing length/length of profemur 3.38–3.58, 3.45.

*Coloration of preserved specimens.* Head and tergites brown. Thorax brown with dark spot.

*Head.* Antenna (Fig. 9) with ten flagellomeres. AR 0.33–0.37. Ultimate flagellomere 108–115, 112 μm long. Temporal seta one, including one outer vertical. Clypeus with 3–4 setae. Tentorium 62–72, 69 μm long, 7–9, 8 μm wide. Palpomere lengths (in μm): 12–14, 13; 24–26, 25; 28–34, 31; 28–36,32; 52–54, 53. L: 5^th^/3^rd ^: 1.61–1.83, 1.81.

**Figures 8–13.**
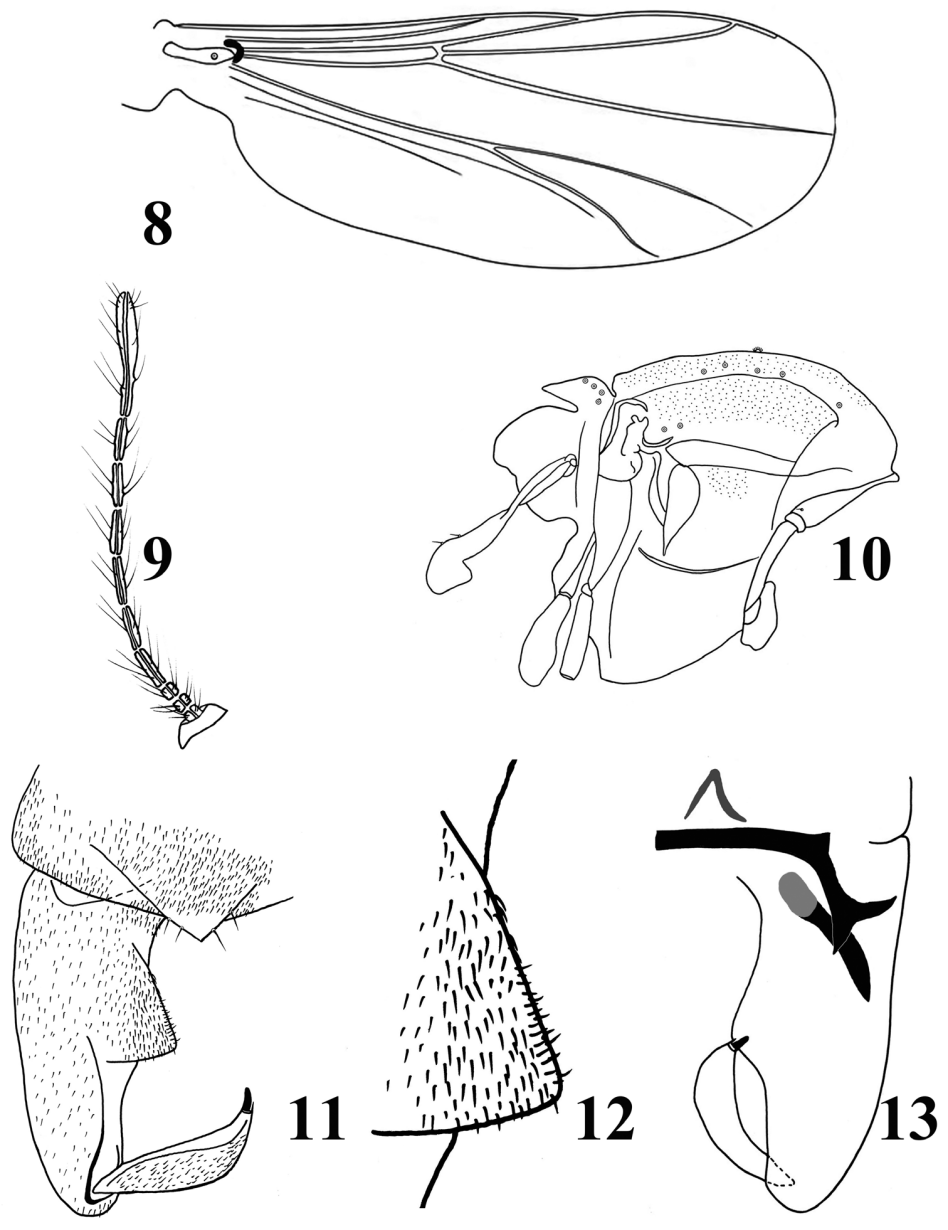
*Parakiefferiella liupanensis* Liu & Wang, sp. n., male. **8** wing **9** antenna **10** thorax **11** hypopygium (dorsal view) **12** inferior **13** hypopygium (ventral view).

*Wing* (Fig. 8). Anal lobe moderate reduced. VR 1.02–1.18, 1.14. Costal extension 40 μm long. Brachiolum with one seta. All of the veins bare. Squama bare.

*Thorax* (Fig. 10). Antepronotal lobes developed. Antepronotum with one lateral seta. Dorsocentrals 5–7, 6; acrostichals absent; two prealars. Scutellum with four setae. Mesonotum with a tuft of hairs.

*Legs*. Spur of fore tibia 12–21, 18 μm long; spurs of mid tibia 9–12, 11 μm and 12–14, 13 μm long; spurs of hind tibia 9–10, 9 μm and 20–22, 21 μm long. Hind tibial comb with 9–12, 10 spines, 19–38, 23 μm long. Width at apex of fore tibia 19–22, 20 mm, of mid tibia 12–22, 17 mm, of hind tibia 18–22, 21 mm. Lengths (in μm) and proportions of legs as in Table 2.

**Table 2.** Lengths (in μm) and proportions of legs of *Parakiefferiella liupanensis* Liu & Wang, sp. n. (n = 5).

	P_1_	P_2_	P_3_
fe	236–242, 239	264–272, 268	239–248, 241
ti	288–302, 298	216–241, 232	252–272, 265
ta_1_	141–153, 145	108–120, 109	132–137, 136
ta_2_	98–116, 111	60–64, 63	72–80, 74
ta_3_	72–76, 74	50–54, 52	74–79, 76
ta_4_	36–40, 38	26–31, 29	36–48, 43
ta_5_	31–36, 34	30–33, 31	33–38, 36
LR	0.49–0.53, 0.51	0.45–0.50, 0.49	0.48–0.52, 0.50
BV	2.62–2.72, 2. 65	3.61–3.66, 3.63	2.85–2.89, 2.87
SV	3.50–3.66, 3.61	4.69–4.77, 4.71	3.73–3.84, 3.79
BR	1.25–1.75, 1.50	2.50–2.75, 2.60	3.40–3.75, 3.60

*Hypopygium* (Figs 11–13). Anal point obtuse triangular with pointed and hyaline apex, lacking keel and bearing 4 setae placed laterally (2 on each side). Laterosernite IX with 2–4, 3 setae. Phallapodeme 19–21, 20 μm long. Transverse sternapodeme, straight 44–48, 46 μm long. Virga consisting of two spines, 20 –25, 23 μm long. Gonocoxite 79–82, 80 μm long. Superior volsella absent; inferior volsella square, occupying about half of the gonocoxite length, lacking dorsal setae and covered with microtrichia (Fig. 12); ventral margin swollen medially. Gonostylus 36–38, 37 μm long, narrowed apically, distinctly bent and curved inward in dorsal view; crista dorsal absent. Megaseta 5–7, 7 μm long. Without crista dorsalis. HR 2.20–2.28, 2.23. HV 2.36–2.43, 2.41.

#### Etymology.

The specific name refers to the Liupan Mountain where the species was collected.

#### Remarks.

The new species can easily be separated from other related *Parakiefferiella* species by the following characters: antenna with 10 segments; anal point square, lacking keel and bearing four setae laterally; inferior volsella without dorsal setae and covered with microtrichia.

*Parakiefferiella liupanensis* Liu & Wang, sp. n. is closely related to *Parakiefferiella bathophila* (Kiffer, 1912). However, the new species differs from the latter on the basis of the following characters: (1) the antenna of the new species with ten flagellomeres, whereas *Parakiefferiella bathophila* possesses 13 flagellomeres; (2) the hypopygium of *Parakiefferiella bathophila* with triangular superior volsella, whereas in the new species it is absent; (3) the anal point of the new species obtuse triangular, not tapered apically, whereas *Parakiefferiella bathophila* gradually tapered to apex; (4) the shape of inferior volsella in the new species square, without dorsal setae, whereas *Parakiefferiella bathophila* triangular, with dorsal setae.

Female and immature stages unknown.

### Parakiefferiella tamatriangulata

Taxon classificationAnimaliaDipteraChironomidae

Sasa,1981

Parakiefferiella tamatriangulata Sasa, 1981: 94; Sasa and Arakawa 1994: 99.

#### Specimens examined.

1 ♂, Liaoning Province, Dandong City, Fengcheng County, 40°51'00"N, 124°07'00"E, 25.iv. 1992, Sweeping net, Wang JC.

#### Diagnostic characters.

Total length 2.06–2.29 mm. Wing length 1.02–1.37 mm. AR 0.38–0.53. Anal lobe reduced. Laterosernite IX with five setae. Phallapodeme 36–38 μm long. Transverse sternapodeme 74–81 μm long. Anal point small, triangular and with pointed apex, bare and hyaline excepting the basal portion with microtrichia. Inferior volsella semicircular, bearing dorsal setae. Gonostylus 6 μm long, narrowed apically, distinctly bent and curved inward in dorsal view. Megaseta 10 μm long. HR 2.18. HV 1.56.

#### Remarks.

The Chinese specimens mainly agree with the original description by Sasa (1981), but they have higher AR (0.53) than the specimens from Japan (0.38–0.48).

#### Distribution.

China (Palaearctic China: Liaoning Province), Japan.

### Key to known adult males of the genus *Parakiefferiella* in China

**Table:** 

1	Tergites banded	**2**
–	Tergites unicolor	**3**
2	Tergites III and VIII dark brown, others pale yellow; all veins bare	***Parakiefferiella fasciata* Liu & Wang, sp. n.**
–	All tergites with a narrow dark band on caudal margin; veins very delicate and colorless	***Parakiefferiella tipuliformis* (Tokunaga)**
3	Antenna with 13 flagellomeres; inferior volsella triangular or semicircular, bearing dorsal setae	**4**
–	Antenna with 10 flagellomeres; inferior volsella square lacking dorsal setae	***Parakiefferiella liupanensis* Liu & Wang, sp. n.**
4	Anal lobe developed; inferior volsella large, roughly triangular	***Parakiefferiella bathophila* (Kieffer)**
–	Anal lobe reduced; inferior volsella semicircular	***Parakiefferiella tamatriangulata* Sasa**

## Supplementary Material

XML Treatment for Parakiefferiella

XML Treatment for Parakiefferiella fasciata

XML Treatment for Parakiefferiella liupanensis

XML Treatment for Parakiefferiella tamatriangulata
